# Giant Hiatus Hernia Causing Pulmonary Vein Obstruction: Insights From 4D Flow Cardiac MRI

**DOI:** 10.1002/ccr3.71567

**Published:** 2025-11-29

**Authors:** Jia Wei Tan, Zia Mehmood, Pankaj Garg

**Affiliations:** ^1^ Norfolk and Norwich University Hospitals NHS Foundation Trust Norfolk UK; ^2^ Norwich Medical School University of East Anglia Norfolk UK

**Keywords:** cardiovascular magnetic resonance, heart failure, hiatus hernia, left atrial compression

## Abstract

Severe extrinsic compression of the left atrium by a giant hiatus hernia can obstruct left atrial inflow. 4D flow cardiovascular magnetic resonance is a novel, non‐invasive imaging technique that can precisely quantify individual pulmonary vein flow dynamics, facilitating the diagnosis of functional venous obstruction in this rare clinical scenario.

A 64‐year‐old woman presented with progressive symptoms of heart failure. A comprehensive cardiovascular magnetic resonance (CMR) study was requested to phenotype a provisional diagnosis of non‐ischemic dilated cardiomyopathy.

The investigation was performed on a Siemens 1.5 T scanner using a protocol including cine imaging, late gadolinium enhancement (LGE), and whole‐heart 4D flow. Initial findings aligned with the referral; the left ventricle was dilated with moderately impaired systolic function (LVEF 39%). Myocardial tissue characterization was unremarkable with no LGE. Overall, CMR supported a diagnosis of dilated cardiomyopathy (DCM).

In addition, the study identified a giant hiatus hernia exerting significant mass effect on the posterior cardiac structures [1]. Axial views showed severe external compression of the left atrium (LA), deforming its posterior wall and deviating the interatrial septum towards the right atrium (Figure 1). This raised suspicion of impaired pulmonary venous (PV) return, prompting 4D flow analysis of individual PV flow. Two key pathologies emerged: first, the right upper, right lower, and left upper pulmonary veins (RUPV, RLPV, LUPV) all showed a diastolic dominant pattern with a blunted systolic wave (S/D ratio < 1), consistent with elevated LA pressure. Second, and more critically, the left lower PV (LLPV) was functionally obstructed by compression. Robustness was confirmed using conservation of mass: total PV inflow (64 mL/cycle) closely matched mitral inflow (66 mL/cycle) and aortic outflow (62 mL/cycle), lending high confidence to the finding of severely redistributed and focally obstructed venous return.

**FIGURE 1:**
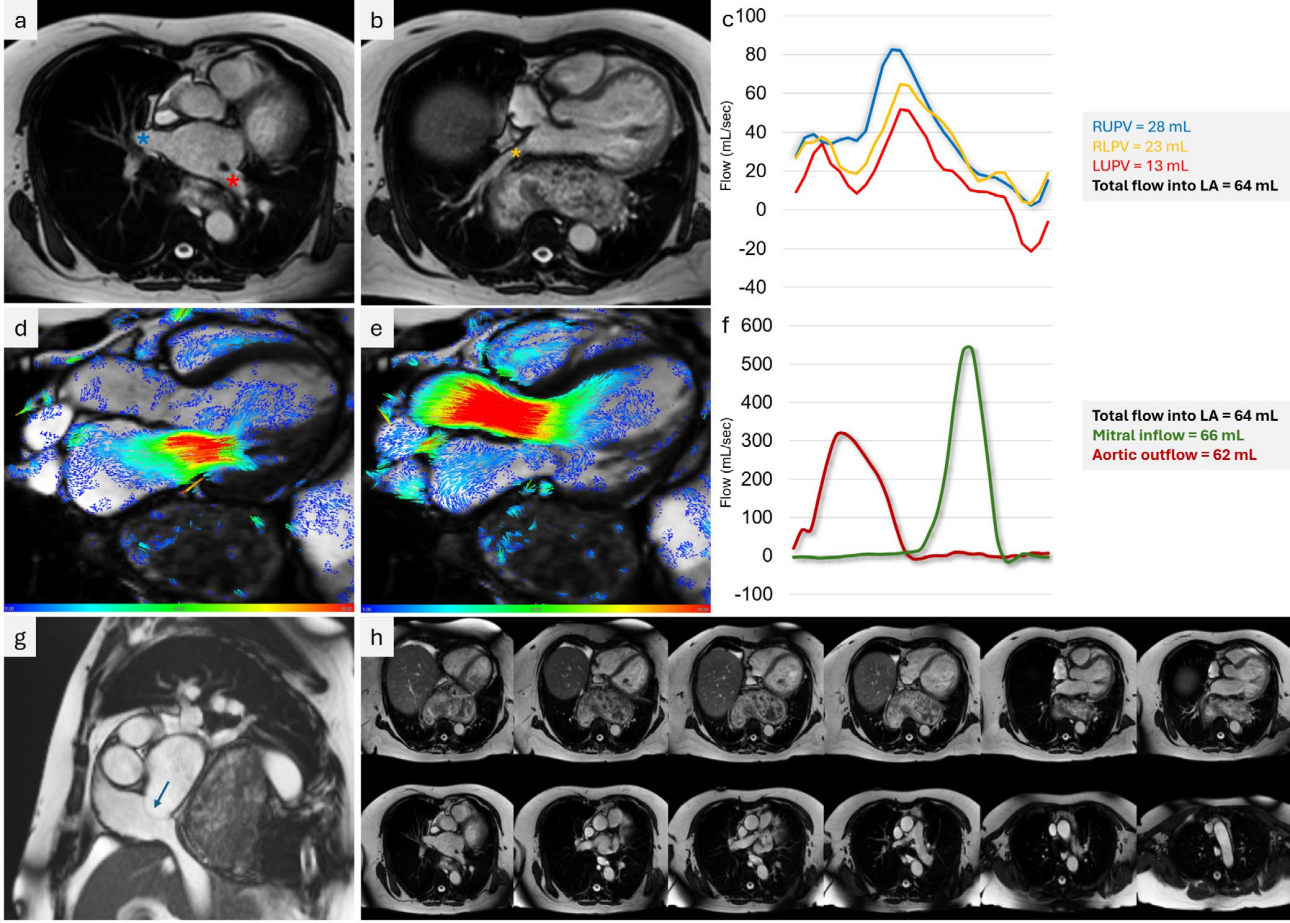
4D Flow CMR and anatomical evaluation of pulmonary veins, mitral/aortic dynamics and hiatal hernia‐mediated left atrial compression. (a) Axial T1‐weighted cardiac MRI at upper pulmonary vein (PV) level showing right upper PV (blue asterisk) and left upper PV draining into the left atrium (LA). (b) Same plane at lower PV level with right lower PV (yellow asterisk) adjacent to the hiatal hernia (HH). (c) Time‐resolved flow curves through RUPV, RLPV and LUPV: D > S pattern (dominant diastolic over systolic flow) consistent with elevated LA pressure. Volumetric return into LA totals 64 mL per cycle. (d–e) 4D‐flow streamline visualizations overlaid on cine imaging showing mitral inflow (d) and aortic outflow (e) over the same timeframe. (f) Flow‐time curves from panels (d–e): Mitral inflow 66 mL, aortic outflow 62 mL (balanced with LA input). (g) Sagittal MRI short‐axis view demonstrating external LA compression by HH (arrow). (h) Axial thoracic stack showing large HH with mediastinal mass effect, impinging on PV and LA anatomy. 4D‐flow MR, four‐dimensional flow magnetic resonance imaging; HH, hiatal hernia; LA, left atrium; LUPV, left upper pulmonary vein; PV, pulmonary vein; RUPV, right upper pulmonary vein; RLPV, right lower pulmonary vein.

This case is highly unusual in that the initial presentation of DCM, which could have led to premature diagnostic closure. It highlights a powerful and novel application of 4D flow CMR, a technique that is increasingly recognized for its ability to circumvent the limitations of conventional imaging [2]. While standard cine imaging identified the anatomical compression, it was the quantitative, non‐invasive assessment of hemodynamics that pinpointed the functional consequence. 4D flow CMR provided the unique ability to dissect the flow from each individual pulmonary vein, confirming high downstream pressure and identifying the functional obstruction of the LLPV.

In conclusion, this case underscores the importance of considering extrinsic cardiac compression in patients with large hiatus hernia. It demonstrates how 4D flow CMR can serve as a critical, non‐invasive tool to move beyond anatomy and provide precise functional data, directly facilitating the diagnosis of rare cardiovascular pathologies. Given the evidence of pulmonary vein obstruction, multidisciplinary discussions are ongoing between cardiology, upper gastrointestinal surgery, and anesthetic teams to determine the optimal strategy, weighing conservative management against surgical repair.

## Author Contributions

**Jia Wei Tan:** conceptualization, data curation, formal analysis, investigation, writing – original draft. **Zia Mehmood:** conceptualization, data curation, project administration, software, writing – original draft. **Pankaj Garg:** resources, supervision, validation, visualization, writing – review and editing.

## Funding

The authors have nothing to report.

## Consent

The authors confirm that written consent for submission and publication of this case report, including images and associated text, has been obtained from the patient in line with COPE guidance.

## Conflicts of Interest

P.G. has a clinical advisor role with Medis Medical Imaging and Pie Medical Imaging. P.G. consults for Edward Lifesciences, Abbott and Anteris.

## Data Availability

The authors have nothing to report.
